# Expression of ammonia transporters Rhbg and Rhcg in mouse skeletal muscle and the effect of 6-week training on these proteins

**DOI:** 10.14814/phy2.12596

**Published:** 2015-10-14

**Authors:** Kohei Takeda, Tohru Takemasa

**Affiliations:** Graduate School of Comprehensive Human Science, University of TsukubaTsukuba, Japan

**Keywords:** Ammonia transporter, skeletal muscle, training

## Abstract

The purposes of our study were to examine (1) Rh B glycoprotein (Rhbg) and Rh C glycoprotein (Rhcg) expressions in mouse skeletal muscle; and (2) the effect of 6-week training on Rhbg and Rhcg expressions. The levels of Rhbg and Rhcg expressions were much higher in the soleus (Sol) than in the plantaris (Pla) or gastrocnemius (Gas). Immunofluorescence microscopy demonstrated that Rhbg colocalizes with dystrophin, a plasma membrane protein marker, whereas Rhcg colocalizes with CD31, a vascular endothelial cell marker. In a 6-week swim training study, we set up two different training groups. Endurance (END) group underwent swim training without load for 30 min and exercise time was increased by 30 min every 2 weeks. High-intensity interval training (HIIT) group underwent 10–12 sets of swim training at 20 sec per set and intervals of 10 sec, with a load of 10% body weight. After 6 weeks of training, all mice performed exhaustive swimming performance test (PT), with 9% of body weight to exhaustion. HIIT group could significantly swim more and showed significantly lower blood ammonia level compared with control (CON) group at immediately after PT. Rhbg and Rhcg levels did not change after 6 weeks in both END and HIIT groups. Our results indicate that ammonia transporters are more abundant in slow fiber than fast fiber muscles and 6 weeks swim training suppresses blood ammonia elevation induced by high-intensity exercise with performance improvement, although the levels of ammonia transporter proteins does not change.

## Introduction

It is well known that exercise-induced fatigue, which is attributable to many factors, including accumulation of metabolites, depletion of muscle glycogen, and so on, affects performance (Bergstrom et al. 1967; Hermansen et al. 1967; Sahlin 1992). Ammonia is a metabolite of several biochemical pathways in the body and is produced in the gut, brain, and kidney (Olde Damink et al. 2002). During exercise, ammonia is produced and accumulates in skeletal muscle when AMP is deaminated to IMP by AMP deaminase (AMPD) during resynthesis of ATP. This may be illustrated by the following equation: 

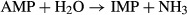


Some studies on animal and human subjects have reported that blood ammonia increased after high-intensity exercise (Barnes et al. 1964; Wilkerson et al. 1977; Itoh and Ohkuwa 1990, 1991; Urhausen and Kindermann 1992). Exhaustive treadmill running has been reported to increase blood ammonia level from 37.8 to 68.1 μmol/L (5). Barns et al. have reported that rats that were forced to swim to exhaustion showed significantly elevated ammonia levels (Barnes et al. 1964). Ammonia is very toxic and has harmful influences on the body, including activation of phosphofructokinase (PFK), which is the rate-limiting enzyme in glycolysis, and inhibition of pyruvate oxidation to acetyl CoA (Katunuma et al. 1966; Lowenstein 1972). Activated PFK facilitates the production of lactate, causing a decline in intercellular pH, decreased release of Ca^2+^ from the sarcoplasmic reticulum, and consequently, a decrease in muscle contractility (Fitts and Balog 1996). Inhibition of pyruvate oxidation hinders the supply of ATP to skeletal muscle, causing fatigue. Moreover, ammonia affects the central nervous system through depletion of glutamate and gamma-aminobutyric acid (GABA), which are both essential neurotransmitters in the brain (Hindfelt et al. 1977; McCandless and Scott 1981; Mutch and Banister 1983). Hence, accumulation of ammonia has an unfavorable effect on exercise tolerance. In the detoxification of ammonia in the liver, the urea cycle is responsible in degrading ammonia to urea (Hirai et al. 1995). Thus, fast ammonia transport from the skeletal muscle to the circulatory system is necessary for its detoxification during exercise. However, the mechanism of its release from the skeletal muscle into the circulation is unclear.

A family of ammonia transporters has been identified in mouse, rat, and human tissues. There are three mammalian members of ammonia transporters, namely, Rh A glycoprotein (Rhag), Rh B glycoprotein (Rhbg), and Rh C glycoprotein (Rhcg) (Liu et al. 2000, 2001; James et al. 2001). Rhag is expressed on the erythrocyte membrane (Liu et al. 1993), whereas Rhbg and Rhcg are widely expressed on plasma membranes of various mammalian organs such as the liver, kidney, and gastrointestinal tract (Weiner and Verlander 2003; Weiner et al. 2003; Handlogten et al. 2005). However, the expression of these proteins in the muscle has not been reported. Nevertheless, skeletal muscle is a major source of ammonia during exercise. Therefore, we hypothesized that ammonia transporters (Rhbg and Rhcg) would be expressed in skeletal muscle. The first aim of this study was to investigate the expression of Rhbg and Rhcg in skeletal muscle based on western blot and immunofluorescence analyses.

It is known that training improves blood ammonia levels during or after exercise. Denis et al. reported that 3 weeks of endurance bicycle training (60–90% VO_2_ max) led to lower blood ammonia levels during 45 min of running compared with blood ammonia levels during pretraining state (Denis et al. 1989). Majerczak et al. also reported that 5 weeks moderate intensity endurance training induced decrease in blood ammonia level during incremental cycling (Majerczak et al. 2012). In addition, 7 weeks of sprint training also reduced blood ammonia levels after 30 sec of maximal exercise in humans (Snow et al. 1992). However, it remains to be clarified whether long-term training improves blood ammonia levels in mice, as demonstrated in humans. Furthermore, the effect of training on ammonia transporter protein expression in skeletal muscle is unclear. Thus, the second aim of this study was to investigate the effects of long-term exhaustive exercise training on blood ammonia level and transporter protein expression in skeletal muscle. Therefore, we carried out two distinct studies to verify each purpose. First, we investigated the characterizations of Rhbg and Rhcg in mouse skeletal muscle. Second, we let mouse 6-week swimming training and exhaustive swimming performance test (PT). Then, exercise performance, blood lactate, ammonia, and Rhbg and Rhcg contents in skeletal muscle were investigated. The hypotheses are that both Rhbg and Rhcg are expressed in skeletal muscle and these proteins content may change by 6 weeks training.

## Material and Methods

### Experiment approval

Animal experiments were carried out in a humane manner after receiving approval from the Institutional Animal Experiment Committee of the University of Tsukuba and in accordance with the Regulations for Animal Experimentation of the university and Fundamental Guidelines for Proper Conduct of Animal Experiments and Related Activities in Academic Research Institutions under the jurisdiction of Ministry of Education, Culture, Sports, Science and Technology of Japan.

### Animals

Eight-week-old male ICR mice were kept in an environment with the temperature of 22 ± 1°C and relative humidity of 60 ± 10% under alternating light and dark cycles of 12 h each, with lights on at 07:00 am. All mice were provided with normal diet (MF, ORIENTAL YEAST Co., Tokyo, Japan) and water ad libitum.

### Tissue preparation

Sol, Pla, and Gas skeletal muscle samples were dissected from the right leg of the subjects, and were quickly frozen using liquid nitrogen and stored at −80°C. Left leg samples were frozen in liquid nitrogen-cooled isopentane. Transverse sections (8 *μ*m) were cut from the mid-belly using cryostat (Microm Cryo-Star HM560, Microm, Walldorf, Germany) and stored at −30°C until analyses.

### Protein content

Sol, Pla, and Gas samples were homogenized in lysis buffer (50 mmol/L Hepes, pH 7.4, 150 mmol/L NaCl, 2 mmol/L EDTA, 1% sodium deoxycholate, 1% NP-40, and 0.2% sodium dodecyl sulfate) with a protease inhibitor mixture (AEBSF, aprotinin, E-64, leupeptin, bestatin, and pepstatin A; Nacalai Tesque Inc., Kyoto, Japan) on ice. Protein concentrations were measured using a Protein Assay Bicinchoninate Kit (Nacalai Tesque Inc.).

### SDS-PAGE MHC isoform analysis

MHC composition was analyzed using SDS-PAGE. After determination of protein content, a sample of denatured myofibrillar protein was loaded onto a 16-cm vertical gel and subjected to electrophoresis. The running gel solution contained 30% glycerol, 8% acrylamide (*N*,*N*′-methylene-*bis*-acrylamide [*bis*] [50:1], 0.2 mol/L Tris–HCl [pH 8.8], 0.1 mol/L glycine), and 0.4% SDS. The stacking gel solution contained 4% acrylamide, 70 mmol/L Tris–HCl (pH 6.7), 4 mmol/L ethylenediaminetetraacetic acid, and 0.4% SDS. Samples were run at a constant voltage (240 V) for about 24 h at 4°C. Gels were stained with Coomassie Brilliant Blue R-250 and destained using methanol–acetic acid solution. Using this system, the MHC IIa and the MHC IId/x bands could not be separated consistently; therefore, the combined signal for both bands was used as a single value, MHC IIa + d/x. Gels were scanned digitally using a computer scanner (GT-9700F, EPSON, Nagano, Japan) and the expression levels of MHC I, IIa + d/x, and IIb were estimated using the NIH Image J program.

### Western blotting

The protein concentration of each homogenized sample, obtained as described above, was adjusted to 2.0 mg/mL with SDS-PAGE loading buffer (62.5 mmol/L Tris–HCl, pH 6.8, 2% w/v SDS, 10% glycerol, 50 mmol/L DTT, and 0.01% w/v bromophenol blue). An equivalent volume of each sample was loaded onto 10% polyacrylamide gel. An electrically blotted PVDF membrane (Bio-Rad, Hercules, CA) was subjected to blocking with 5% skim milk in TBS containing 0.05% Tween 20 (TBST) for 1 h at room temperature. Antibodies against Rhbg and Rhcg (Abcam, Cambridge, UK) were diluted 1:3000 with blocking buffer and used as the primary antibodies and incubated for 12 h at 4°C. Anti-goat or anti-rabbit IgG-conjugated HRP (Life Technologies, Carlsbad, CA) was diluted 1:10,000 with blocking buffer and used as the secondary antibody and incubated for 1 h at room temperature. After carefully washing with TBST, bound antibody complexes were visualized using Chemi-Lumi One Super (Nacalai Tesque Inc.) and VersaDoc 5000 (Bio-Rad, Hercules, CA). The stained PVDF membranes were analyzed using the NIH Image J program.

### Immunohistochemical analysis

Frozen sections were fixed in 4% paraformaldehyde in PBS and were made permeable using 0.5% Triton-X100 in PBS. All subsequent washes were performed with 0.1% Tween 20 in TBS. Sections were blocked in 5% horse serum for 60 min, followed by incubation overnight at 4°C with diluted primary antibody anti-Rhbg (1:200; Abcam, Cambridge, UK), anti-Rhcg (1:200; Abcam), anti-dystrophin (1:100; Abcam), and anti-CD31 (1:300; BioLegend, San Diego, CA). Secondary antibodies were Alexa Fluor 546-conjugated goat anti-rabbit IgG (1:300; Life Technologies), Alexa Flour 488-conjugated donkey anti-mouse IgG (1:300; Abcam), and Cy3-conjugated donkey anti-goat (1:300; Jackson ImmunoResearch, West Grove, PA) with DAPI (Life Technologies). Subsequently, the sections were incubated for 60 min at room temperature. After several washes, the sections were enclosed on a mounting medium (KPL, Gaithersburg, MD). The images were obtained under a microscope (BX-51, Olympus, Tokyo, Japan) and processed using image analysis software (Openlab, PerkinElmer, Waltham, MA).

### Training protocol

**Figure 1:**
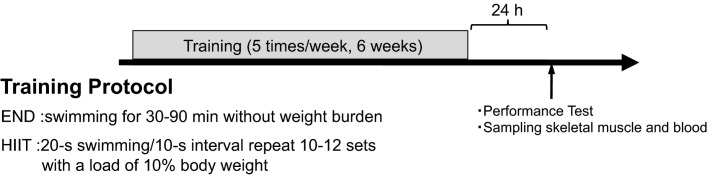
Schema of experimental design. Thirty-six mice were divided into three groups: control (Con), endurance training (END), and high-intensity interval training (HIIT). END and HIIT groups performed each training protocol for 6 weeks. Twenty-four hours after last training session, half mice of the each group performed performance test (PT). The mice swam to exhaustion with a load of 9% body weight to their tails. Immediately after PT, blood samples were collected for analysis of lactate and ammonia. Tibials anterior were also collected for muscle glycogen analysis. Not undergoing PT mice (remain of each group) were killed to collect blood samples for sedentary state ammonia and skeletal muscle for western blot and muscle glycogen in the same day.

Thirty-six mice were divided into three groups (*n *=* *12 per group): one control (CON) group and two training groups, END or HIIT. END and HIIT groups underwent swim training five times per week for 6 weeks in a tank (42 × 64 × 38 cm) filled with 30-cm deep water which was kept at 30 ± 1°C. END group swam without any weight burden for 30 min for 2 weeks, with progressive prolongation of exercise time by 30 min every 2 weeks. HIIT group repeated 10 sets of 20-sec swimming at 10-sec intervals with a load of 10% body weight attached to their tails. HIIT training sets were increased by two sets every 2 weeks, with the last 2 weeks having 12 sets. Levels of blood lactate of the training groups were measured every week using Lactate Pro 2 (Arkrey, Tokyo, Japan) before and after training from same mouse. Blood sample (∼0.3 µL) was collected from tail incision by scalpel. Schema of experimental design is showed in Figure1.

### Swimming performance test

After 6 weeks of training, half of each group was subjected to swimming performance test (PT). The mice swam to exhaustion with a load of 9% of body weight attached to their tails. Each mouse was considered to have reached exhaustion when it failed to surface the water for 5 sec for inhalation. Time to exhaustion was defined as swimming performance.

### Blood and muscle sampling

Immediately after exhaustion, the mice were killed by cervical dislocation and blood samples, together with the Sol, Pla, Gas, and tibialis anterior (TA), were collected. Blood samples were collected from the tail by scalpel for lactate and from the heart for ammonia measurements. Muscle samples were stored at −80°C until used. The mice that did not undergo PT were killed 24 h after the last training session; the blood sample, Sol, Pla, Gas, and TA were collected later. The blood samples were used for sedentary state blood ammonia. Sol, Pla, and Gas were used for western blotting analysis. The TA samples were used for sedentary state muscle glycogen content analysis.

### Blood ammonia assay

Blood ammonia was assayed using a commercial kit (Ammonia Test Wako, Wako, Osaka, Japan). Whole blood sample was used in the lactate and ammonia analyses. Blood for lactate analysis was from mouse’s tail by a razor. Lactate Pro 2 can measure lactate ∼0.3 µL whole blood. Blood for ammonia analysis was sampled from the heart about 1 mL. Blood sample (1.0 mL) was immediately mixed with deproteinizing reagent (4.0 mL) and centrifuged at 600 *g* for 5 min at 4°C. Supernatant (2.0 mL) was transferred to the new tube and added the color reagent solution A (2.0 mL). After well shake, added the color reagent solution B (1.0 mL) and the color reagent solution C (2.0 mL). These processes were kept at on ice condition. The sample was then incubated in the water bath at 37°C for 20 min. Absorbance was measured using the micro plate reader (SH-1000, CORONA ELECTRIC Co., Ibaraki, Japan). We used unperformed PT group’s blood samples as Pre (sedentary) and immediately after PT blood samples were used as Post.

### Muscle glycogen assays

Skeletal muscle glycogen concentrations were measured following phenol–sulfuric acid method, as described by Lo et al. (Lo et al. 1970). Muscle samples (10–20 *μ*g) were incubated in 30% KOH + Na_2_SO_4_ at 100°C for 10 min, then at room temperature for 10 min, followed by addition of 360 *μ*L of 100% ethanol before centrifuge. Samples were then incubated on ice for 30 min then centrifuged at 3300 *g* for 20 min at 4°C. The supernatant was separated and poured out and the sample tubes were air dried. The pellets were dissolved in ultrapure water. The sample was mixed with 5% phenol and H_2_SO_4_; subsequently, absorbance was measured using spectrophotometer (UV-1600, SHIMADZU, Kyoto, Japan).

### Statistical analysis

All results were presented as mean ± SE. Blood lactate, blood ammonia, and muscle glycogen concentrations were analyzed using two-way ANOVA and Tukey’s post hoc test. Western blot data and exercise time during PT were analyzed using one-way ANOVA and Tukey’s post hoc test. Correlations were analyzed with the Pearson’s correlation test. The significant level was set at *P *<* *0.05.

## Results

### MHC composition

Sol contained MHC I (38.9 ± 2.8%) and IIa + d/x (61.1 ± 2.8%), which means that this muscle is composed of predominantly slow fibers. Pla and Gas were composed of MHC IIa + d/x (36.2 ± 2.6%, 25.8 ± 3.0%, respectively) and IIb (63.8 ± 2.6%, 74.1 ± 3.0%, respectively), which means these muscles are fast-type dominant (no figure).

### Rhbg and Rhcg expressions

**Figure 2:**
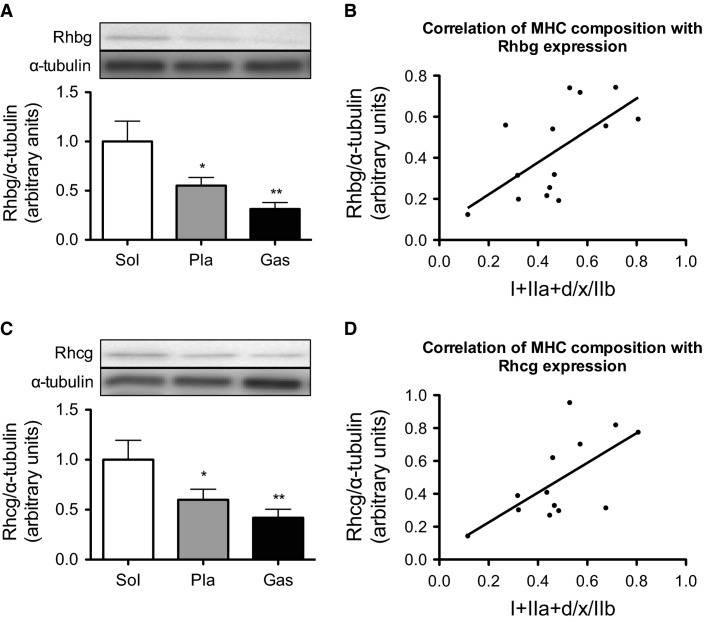
Content of Rhbg (A) and Rhcg (C) in Sol, Pla, and Gas. Values represent the mean ± SE (*n* = 6–7). *P <* *0.05, **P <* *0.01 versus Sol. (B) Correlation between MHC I and IIa + d/x to MHC IIb ratios and Rhbg content in Pla and Gas. (D) Correlation between the MHC I and IIa + d/x to MHC IIb ratios and Rhcg content in Pla and Gas.

To investigate whether the Rhbg and Rhcg are expressed in the skeletal muscle tissue, skeletal muscle samples were collected from sedentary mouse. Rhbg expression was detected by western blotting and is shown in Figure2A. Sol contained more Rhbg than Pla or Gas. A strong positive correlation (*r *=* *0.64, *P *<* *0.05) was found between the MHC composition (I + IIa + d/x)/IIb and Rhbg expression (Fig.2B). Furthermore, Sol had higher Rhcg expression than Pla or Gas (Fig.2C). A similar correlation was observed between MHC composition (I + IIa + d/x)/IIb and Rhcg expression in Pla and Gas (*r *=* *0.64, *P *<* *0.05) (Fig.2D).

### Rhbg and Rhcg localization

**Figure 3:**
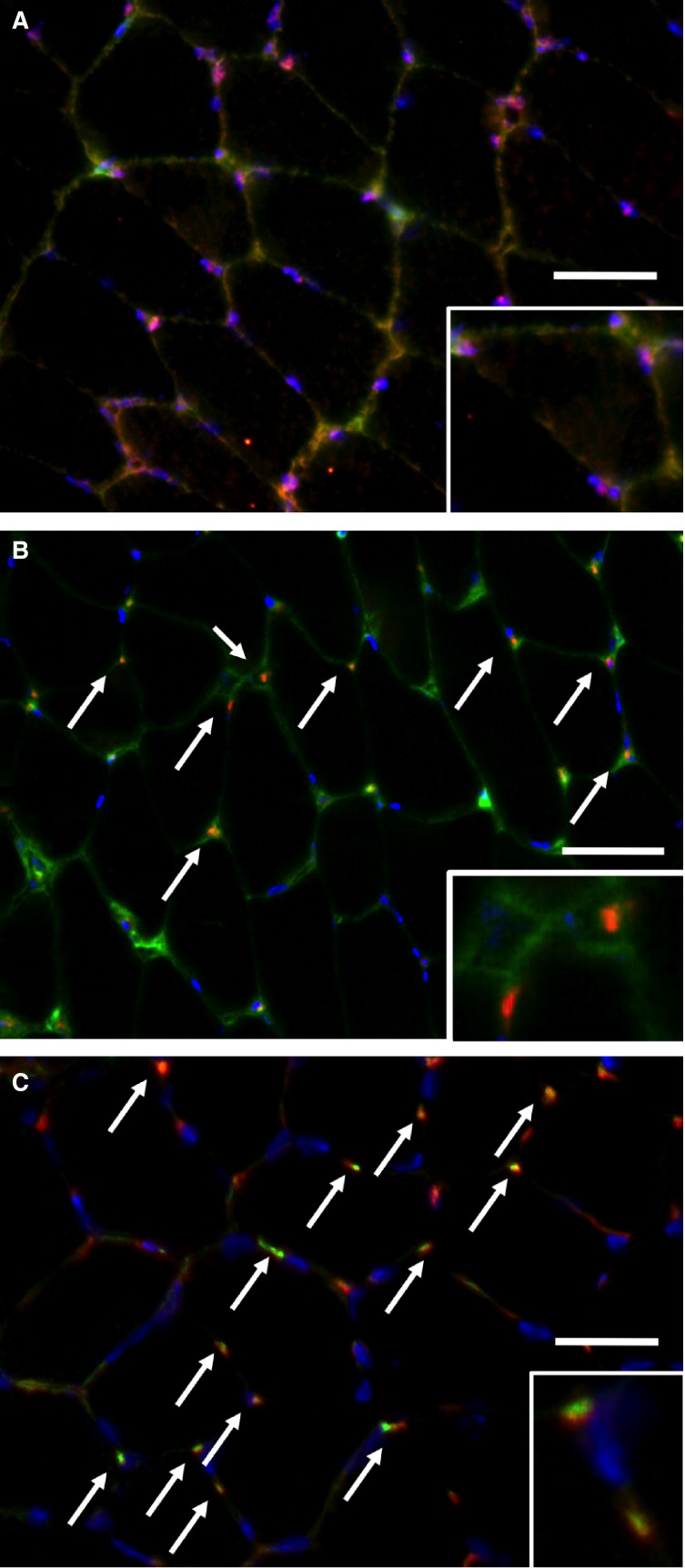
Immunofluorescence analysis of the cellular localizations of ammonia transporters, Rhbg and Rhcg, in skeletal muscle with magnified views (inset). (A) Costaining image of Rhbg (red) and dystrophin (green) with DAPI (blue). (B) Costaining image of Rhcg (red) and dystrophin (green) with DAPI (blue). (C) Costaining image of Rhcg (red) and CD31 (green) with DAPI (blue). Bar = 0.1 mm.

We analyzed the localization of ammonia transporters Rhbg and Rhcg in skeletal muscle using immunofluorescence microscopy. Rhbg colocalized with dystrophin, a plasma membrane protein marker (Fig.3A). Rhcg-positive stain was observed as dots, which did not colocalize with dystrophin (Fig.3B); however, it colocalized with CD31, a vascular endothelial cell marker (Fig.3C). Thus, Rhbg and Rhcg had different localizations in skeletal muscle.

### Blood lactate during training period

**Figure 4:**
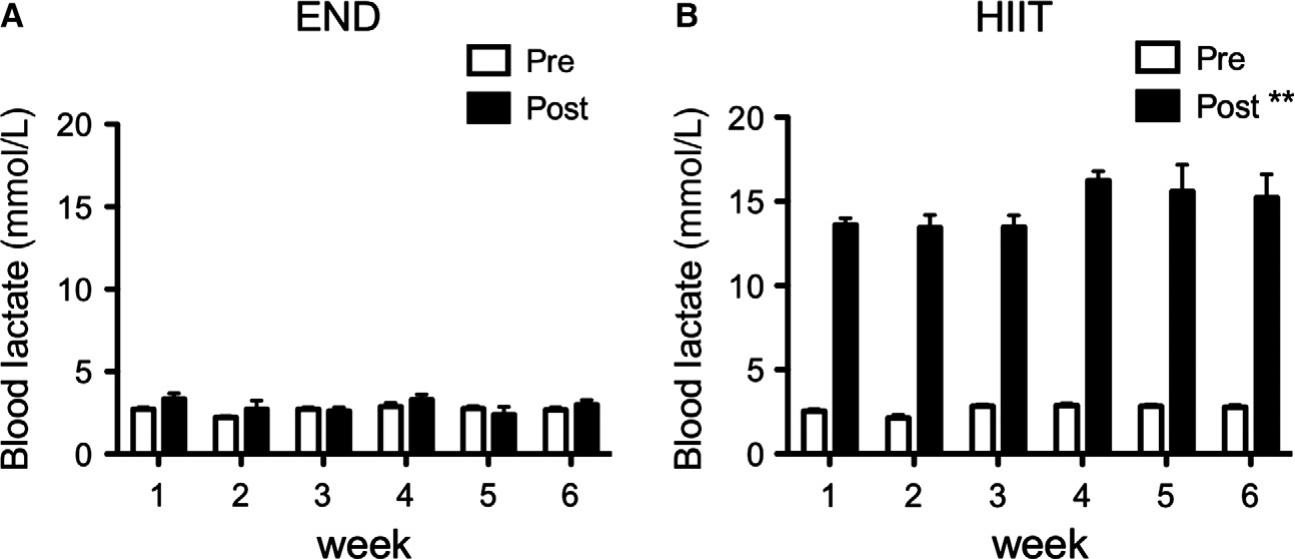
Blood lactate levels during the training period in END (A) and HIIT (B) groups. Blood lactate was measured per week before and after training session from tail. Values represent mean ± SE (*n* = 12). **P <* *0.01 versus pretraining in the same week.

We measured blood lactate every week over the 6-week training period. END group showed no change in blood lactate after exercise, whereas HIIT group demonstrated lactate levels of more than 14 mmol/L after every week of exercise (Fig.4). Both groups performed the same exercise intensity for 6 weeks.

### Blood lactate, ammonia, and muscle glycogen after swimming performance test

**Figure 5:**
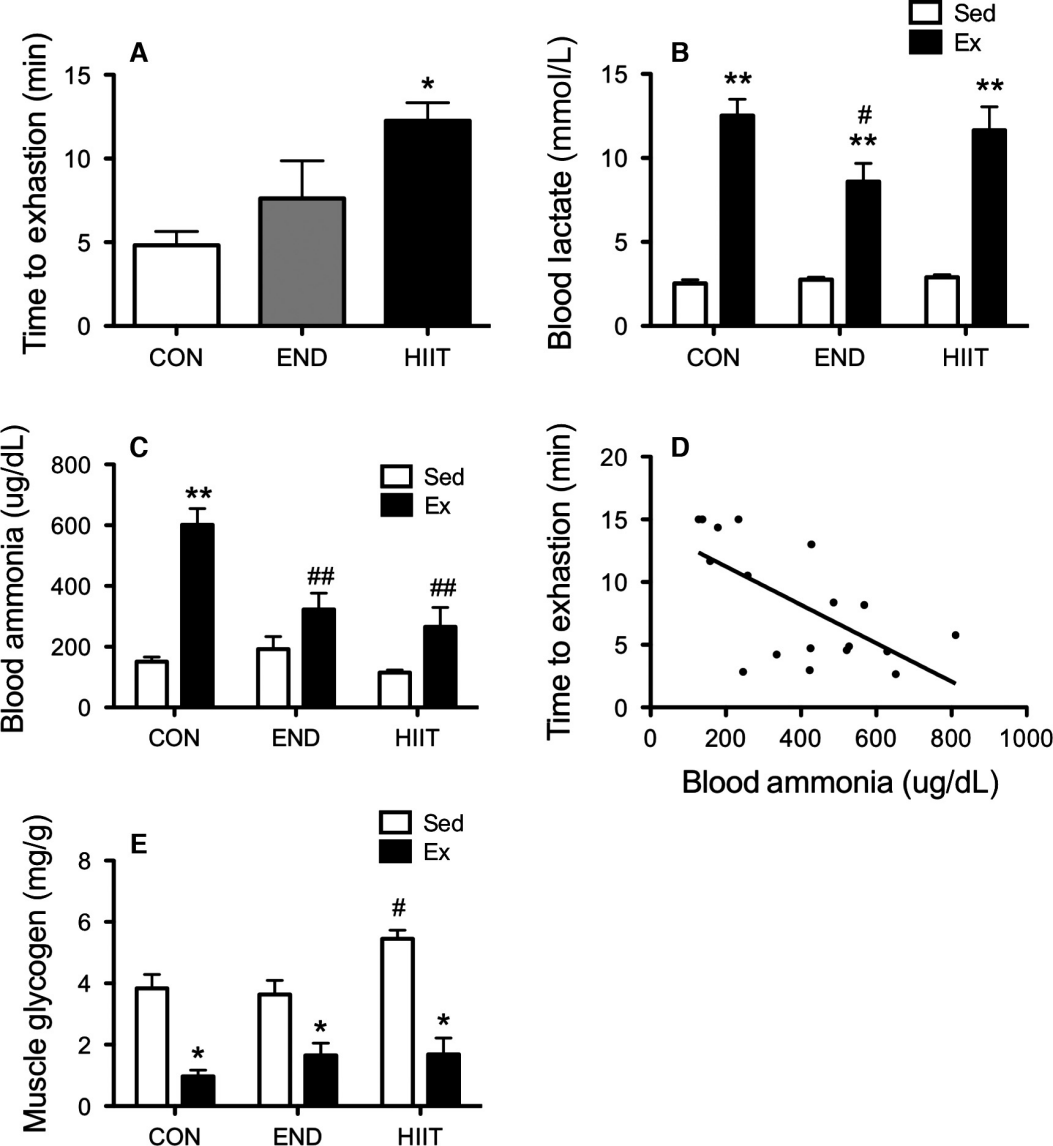
Half of each group mice performed performance test (PT) until exhaustion. (A) The value of exercise time until exhaustion. Values represent mean ± SE. *P <* *0.05 versus CON. Blood lactate (B) and blood ammonia (C) levels before and after PT. Values represent mean ± SE. **P <* *0.01 versus Sed; ^#^*P *<* *0.05, ^##^*P* < 0.01 versus CON. (D) Relationship between blood ammonia level immediately after PT and exercise time to exhaustion. (E) Muscle glycogen content in TA muscle. Values represent mean ± SE. *P <* *0.05 versus Sed; ^#^*P *<* *0.05 versus CON.

Upon evaluation of the PT, HIIT and END groups showed prolongation in the swim time to exhaustion as compared with that of CON group (Fig.5A). All groups had significantly increased blood lactate after exhaustive swimming, whereas END group showed significantly low levels (Fig.5B). Blood ammonia also significantly increased after exhaustive swimming in all groups (Fig.5C). However, both END and HIIT groups showed significantly lower levels of ammonia compared with CON group. There was a significant negative relationship between blood ammonia level and the exercise time to exhaustion (*r* = −0.65, *P *<* *0.01) (Fig.5D). All groups had decreased muscle glycogen contents in TA after PT (Fig.5E). HIIT stored more muscle glycogen than other groups at a sedentary state.

### Protein expression of ammonia transporters Rhbg and Rhcg

**Figure 6:**
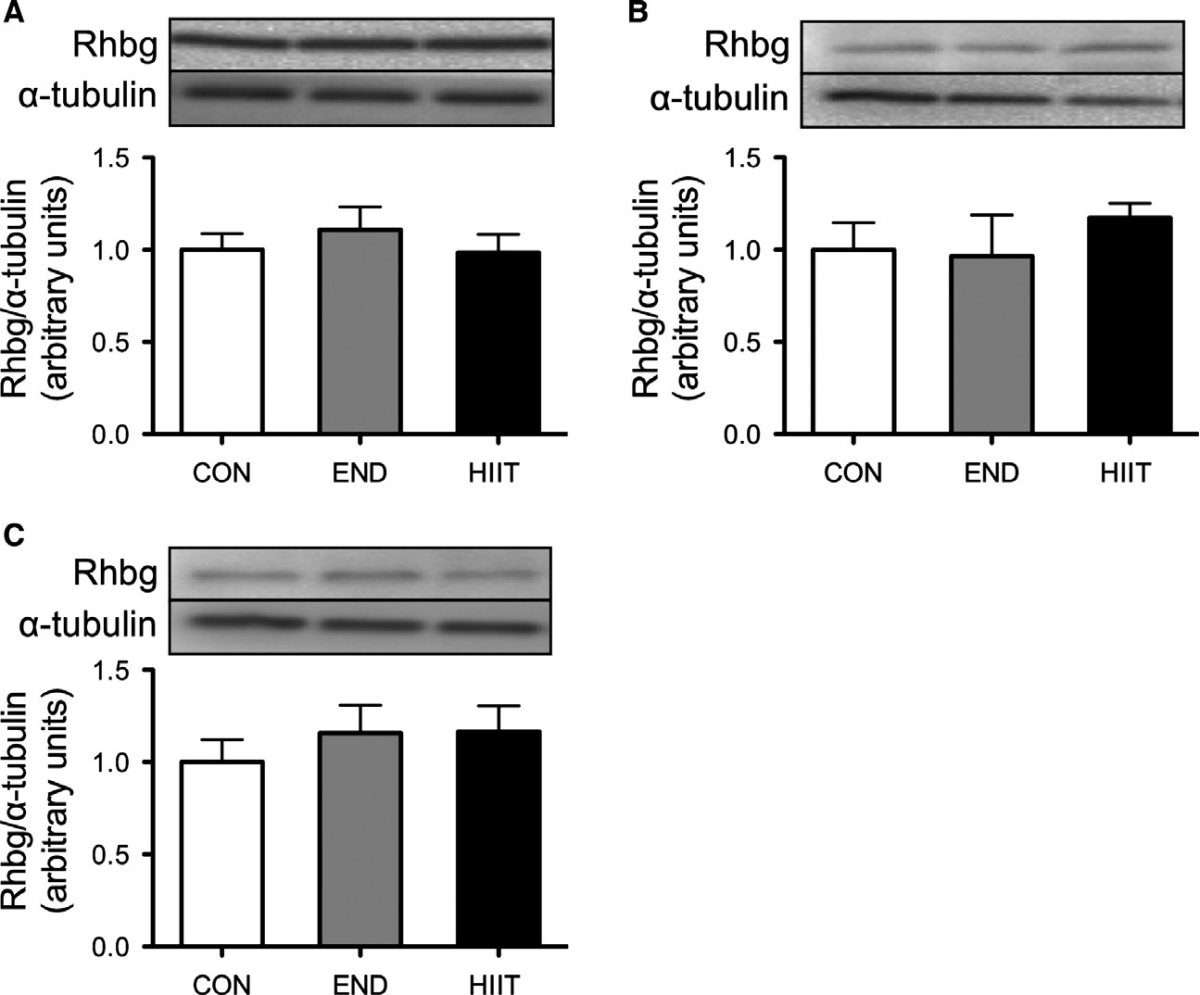
Effect of 6-week training on Rhbg content in the control group (Con), endurance training group (END), and high-intensity interval training group (HIIT). (A) Sol, (B) Pla, and (C) Gas. Values represent mean ± SE (*n* = 6).

**Figure 7:**
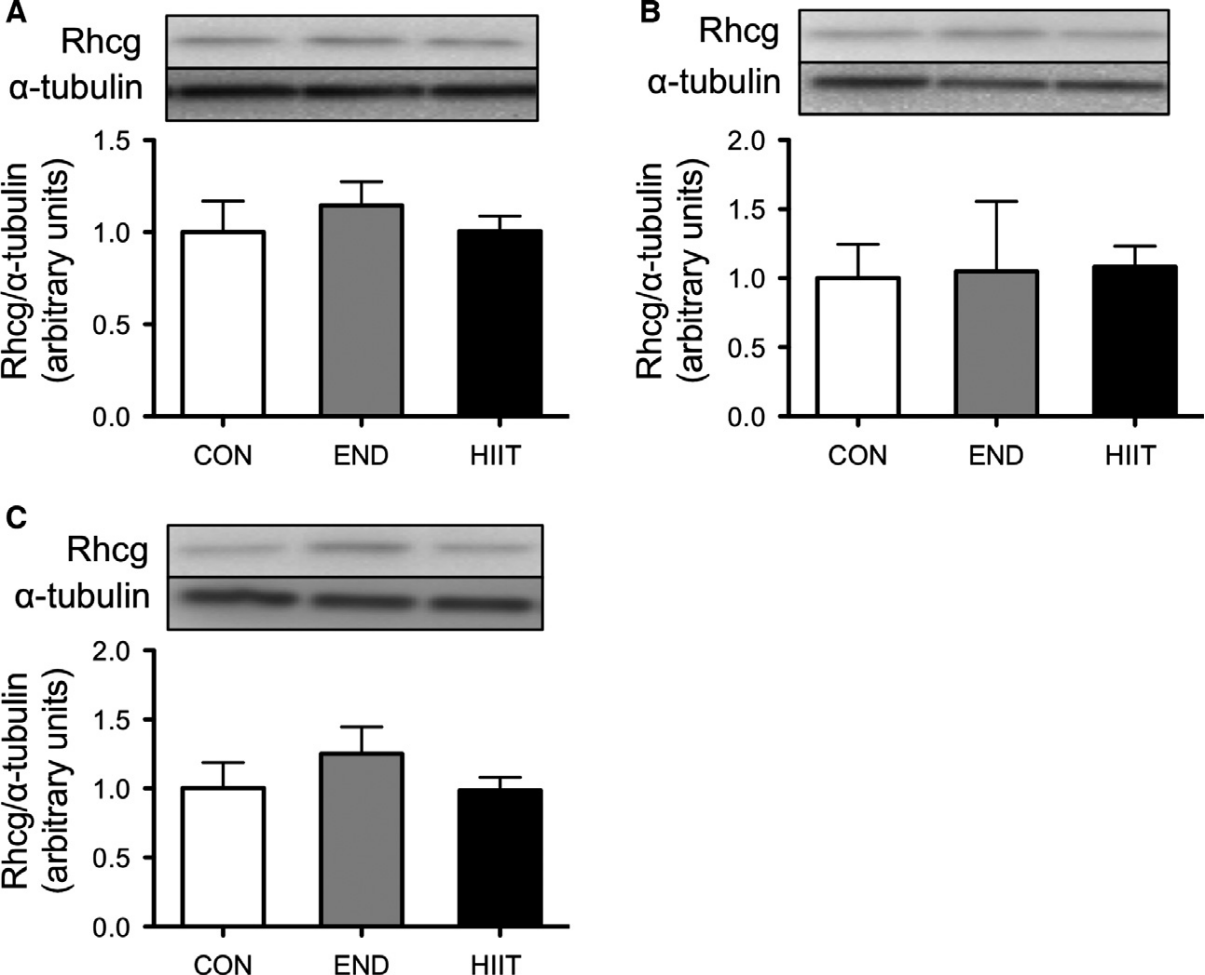
Effect of 6-week training on Rhcg content in the control group (Con), endurance training group (END), and high-intensity interval training group (HIIT). (A) Sol, (B) Pla, and (C) Gas. Values represent mean ± SE (*n* = 6).

The protein content of Rhbg did not significantly change after 6 weeks of END or HIIT in all skeletal muscle groups (Fig.6). Rhcg expression was likewise not significantly different before and after the 6-week training period (Fig.7).

## Discussion

Ammonia is produced via AMP deamination by AMPD in skeletal muscle and is delivered to the bloodstream to be transported to the liver, where it is quickly detoxified to urea in the urea cycle (Hirai et al. 1995). Ammonia accumulation leads to peripheral and central fatigue and affects exercise performance. Because skeletal muscle is the main source of ammonia production via AMP deamination in ATP resynthesis during exercise, elimination of ammonia is particularly important for fast fibers (Winder et al. 1974). However, the mechanism of ammonia release from the muscle to the blood is unclear. To elucidate this process, we focused on the expression of ammonia transporters, Rhbg and Rhcg, in skeletal muscle by using biochemical and immunohistochemical methods. We found that both Rhbg and Rhcg were expressed in mouse skeletal muscle. Western blot analysis showed that both ammonia transporters were abundant in muscles with predominantly slow fiber (Sol) as compared to muscles with predominantly fast fiber (Pla and Gas) (Fig.2). These results may suggest that ammonia transporter activity contributes more to the function of slow fiber than fast fiber muscles. It is not immediately obvious why the expression of ammonia transporters is higher in slow fiber dominant muscles, even though more ammonia is produced during exercise in fast fiber muscles. We speculate that slow fiber muscles have high ammonia intake capacity. Acute liver failure such as toxic liver injury or viral hepatitis leads to dysfunction in ammonia detoxification. Chatauret et al. have reported that the skeletal muscle capacity for ammonia removal increased in acute liver failure (Chatauret et al. 2006). It is possible that ammonia transporters mediate this adaptation. Ammonia produced in fast fiber muscle by physical exercise may be taken into slow fiber muscle via Rhbg and Rhcg. Further studies are needed to verify the detailed functions of these proteins in skeletal muscles.

The different cellular localizations of Rhbg and Rhcg indicate that these ammonia transporters may have different roles in the skeletal muscle (Fig.3). In the mouse liver, basolateral Rhbg is expressed by perivenous hepatocytes, where it may mediate ammonium uptake. However, mouse liver Rhcg is expressed in the bile duct epithelial cells, where it may be involved in ammonium secretion (Weiner et al. 2003). Another study has reported that the Rhbg and Rhcg distribution is different in the mouse kidney, with basolateral expression of Rhbg and apical expression of Rhcg (Weiner and Verlander 2003). These results indicate that Rhbg and Rhcg complement each other in the mouse kidney. Differences in patterns of Rhbg and Rhcg expressions between the liver and kidney suggest possible important variations in ammonia metabolism in the skeletal muscle.

The second aim of this study was to investigate the effect of 6 weeks of training on blood ammonia level after exhaustive exercise and on changes of ammonia transporters expression in skeletal muscle. The 6-week HIIT enhanced exercise performance and both END and HIIT depressed blood ammonia level after complete exercise. Subsequently, we found a significant negative relationship between blood ammonia level and exercise performance (Fig.5D). Continuous endurance training suppressed elevation of blood ammonia after prolonged exercise in humans (Denis et al. 1989). Sprint training has likewise reduced blood ammonia levels (Snow et al. 1992). These results suggest that suppression of ammonia during exercise may enable progression of exercise performance. On the other hand, the molecular mechanism of ammonia metabolism in skeletal muscle has not been investigated. Both END and HIIT for 6 weeks did not affect the expression of Rhbg and Rhcg (Figs.7). The reasons for decreased ammonia level after PT in trained groups is assumed to be change in the energy metabolism for ammonia production in skeletal muscle. Dudley et al. have showed that the higher mitochondria muscle induced by training showed lower ammonia production by stimulating tetanically lower hindlimb compared with untrained muscle. In addition, the levels of AMP and IMP in the muscle were also lower in the trained muscle (Dudley et al. 1987). These results suggest that training-induced biogenesis of mitochondria induced increasing oxidative capacity, which caused the lower ammonia production. Green et al. reported that 5–7 days endurance training induced suppression of glycogen usage during endurance exercise without change of mitochondria capacity and muscle IMP was significantly lower during exercise compared with untrained muscle (Green et al. 1992). In addition, HIIT increases oxidative metabolism capacity, for instance, mitochondria biogenesis and enzyme activity, similar to what END training does (Terada et al. 2001; Ogura et al. 2005; Burgomaster et al. 2008; Gibala and McGee 2008). HIIT also induced decline of AMP deaminase that is associated with ammonia formation (Hellsten-Westing et al. 1993). These reports exhibit that the adaptation of whole energy metabolism may change to supply sufficient ATP without ammonia production during exercise.

To the best of our knowledge, this is the first study to identify the expression and cellular localization of ammonia transporter proteins, Rhbg and Rhcg, in mouse skeletal muscle. Our results showed that Rhbg and Rhcg are abundant in slow fiber muscle and that cellular localizations of these transporters are different. Six weeks of endurance and high-intensity interval training had effects on exercise performance and blood ammonia levels after exhaustion. However, the expression of both ammonia transporters was not affected by long-term training. Further studies are needed to investigate the exact function of Rhbg and Rhcg in the skeletal muscle and their relationship with exercise performance.

## Conflict of Interest

None declared.
